# Whole Genome Sequencing of *Mycobacterium bovis* Isolated From Livestock in the United States, 1989–2018

**DOI:** 10.3389/fvets.2018.00253

**Published:** 2018-10-30

**Authors:** Kathy Orloski, Suelee Robbe-Austerman, Tod Stuber, Bill Hench, Mark Schoenbaum

**Affiliations:** ^1^U.S. Department of Agriculture, Animal and Plant Health Inspection Service, Veterinary Services, Centers for Epidemiology and Animal Health, Fort Collins, CO, United States; ^2^National Veterinary Services Laboratories, U.S. Department of Agriculture, Animal and Plant Health Inspection Service, Veterinary Services, Ames, IA, United States; ^3^U.S. Department of Agriculture, Animal and Plant Health Inspection Service, Veterinary Services, Fort Collins, CO, United States

**Keywords:** *Mycobacterium bovis*, bovine tuberculosis, genotyping, whole genome sequencing, bovine, cervid

## Abstract

The United States official bovine tuberculosis (bTB) eradication program has utilized genotyping for *Mycobacterium bovis* isolates since 2000 and whole genome sequencing was implemented in 2013. The program has been highly successful, yet as bTB prevalence has reached historic lows, a small number of new bTB-affected cattle herds occur annually. Therefore, understanding the epidemiology of bTB transmission is critically important, in order to target limited resources for surveillance and achieve eradication. This evaluation described the diversity and epidemiology of *M. bovis* isolates identified in the USA livestock. Isolates from animals within the bTB endemic area of Michigan were excluded. Broad diversity was found among 1,248 isolates, collected from affected cattle and farmed cervids herds and fed cattle during 1989–2018. Nearly 70% of isolates from 109 herds/cases during 1999–2018 were European clonal complex 1 and 30% were European clonal complex 2. The sources of infection based on the herd investigation were known for 41% of herds/cases and 59% were not epidemiologically linked to another USA origin herd. Whole genome sequencing results were consistent with the investigation findings and previously unrecognized links between herds and cases were disclosed. For herds/cases with an unknown source of infection, WGS results suggested several possible sources, including undocumented cattle movement, imported cattle and humans. The use of WGS in new cases has reduced the time and costs associated with epidemiological investigations. Within herd SNP diversity was evaluated by examining 18 herds with 10 or more isolates sequenced. Forty percent of isolates had not diverged or accumulated any SNPs, and 86% of the isolates had accumulated 3 or fewer SNPs. The results of WGS does not support a bTB reservoir in USA cattle. The bTB eradication program appears to be highly effective as the majority of herds/cases in the USA are unique strains with limited herd to herd transmission.

## Introduction

*Mycobacterium bovis* (bTB) has a broad host range, causing economic loss to beef and dairy production and infecting humans and wildlife. Therefore, most developed countries and many developing countries have national bovine tuberculosis eradication programs in livestock. The United States (USA) began a national eradication program for *M. bovis* infection in cattle in 1917. At the program's inception, the apparent prevalence of bTB was 5% of cattle, as estimated by positive responses to the caudal fold tuberculin skin test (CFT) (1). The program's history has been documented elsewhere, including the reduction of prevalence in cattle to <0.005% of cattle herds today (2, 3).

During 2001 to 2011, 92 U.S. cattle herds were infected with *M. bovis*, in an estimated cattle population of 87 million head on 913,000 operations (3). During 1991–2004, there were 41 bTB-affected farmed cervid herds (4, 5). State and Federal veterinarians conduct extensive investigations when bTB is detected; routinely investigating animals that arrived and left the herd within the last 5 years. The program's cornerstone activities are national surveillance for cattle, bison and farmed cervids and quarantine of bTB-affected herds until the infection is no longer detected in individual animals. Despite these efforts, each year there are 2–15 affected cattle herds (3). Affected farmed cervid herds occur sporadically, with the most recent occurrence in 2009. As the USA bTB prevalence has reached historic lows, understanding the epidemiology of bTB transmission is critically important, in order to target limited resources for surveillance and achieve eradication. Challenges to the final eradication of bTB in the USA include a wildlife reservoir in white-tailed deer in northeastern Michigan, sporadic occurrences in dairies and beef herds, bTB in imported feeder cattle, and limitations in the ability to trace animals (3, 6).

Genotyping of *M. bovis* isolates has been in use since 2000 in the official USA bTB eradication program, beginning with IS*6110* based restriction fragment length polymorphism analysis, then adding spoligotyping in 2004 and multiple loci variable number tandem repeat analysis in 2008. These results showed that strains in the USA were highly diverse in both genotypes and geographical locations, with overlap in strains between USA origin and Mexican origin cattle. However, the low resolution of these genotyping methods failed to identify transmission paths (7). Whole genome sequencing is useful at elucidating sources of infection, resolving indistinguishable genotypes identified by other methods and potentially estimating when a new strain was introduced (8, 9). Whole genome sequencing (WGS) was implemented at NVSL on an experimental basis in 2012 and for official program use in January 2013, when WGS replaced traditional spoligotyping and VNTR. The laboratory was able to provide WGS results within the same time frame as traditional genotyping (typically within 4–6 weeks from tissue submission), which was then used to inform the field investigation. Several training programs and webinars were done to prepare the staff for interpreting results (10).

The objectives of this paper are to characterize *M. bovis* isolates identified in the USA from livestock and captive/farmed wildlife, and describe the molecular epidemiology of *M. bovis* in bTB-affected cattle herds in the USA. This information will assist animal health officials and the cattle industry in understanding the transmission of *M. bovis* and use this information for disease prevention.

## Methods

### Isolate selection

Because all official bTB eradication program laboratory diagnostics were performed at the United States Department of Agriculture (USDA), National Veterinary Services Laboratories (NVSL), all isolates archived and maintained at that facility up to May 2018 were sequenced. Prior to 2000, there were no procedures in place to permanently archive isolates, consequently a limited number of isolates prior to 2000 were available. After 2000, nearly all *M. bovis* isolates that were obtained through official bTB program activities from livestock and other animals residing on premises with bTB-infected cattle or farmed cervids were available. In addition, *M. bovis* and *M. caprae* isolates originating from clinical specimens that were submitted to the NVSL were included. Clinical specimens are defined as those originating from diagnostic submissions from animals that are not legally covered by official bTB eradication program regulations. For example, animals in zoological collections or laboratory animals. These isolates originated from other domestic and captive animals residing in the USA that were not under bTB eradication program regulatory authority. Official USDA records detailing the epidemiological investigations that occurred during federal fiscal years 1999 through June 2018 were correlated with the corresponding sequenced isolates. Because a complete list of confirmed bTB-affected herds was not available prior to 1999, results for isolates identified before 1999 were analyzed separately within this paper. Also included in the paper were five reference isolates, AF2122/97 (Biosample: ERS1462286), Ravenel (Biosample: SAMN04448492), BCG (Biosample: SAMN06847294), AN5 (Biosample: SAMN04448491), and 94-1MIDNRdeerAlp (Biosample: SAMN04386752) (the index Michigan deer isolate). See Supplemental File 1 for isolate metadata. All samples were collected and tested under the authority of the Code of Federal Regulations (CFR) enacted to guide the State-Federal Cooperative bovine tuberculosis eradication program as outlined in 9 CFR part 49, 50.

### Official program standards

Official program activities include ongoing slaughter surveillance, live animal testing, and investigations of bTB-affected herds. These activities are described in the USDA, Bovine Tuberculosis Eradication, Uniform Methods and Rules (11), and are summarized elsewhere (8, 9, 12, 13). Briefly, antemortem testing is performed on cattle, bison and farmed cervids for a number of reasons, such as entry to a show or sale, state entry requirements, and as part of bTB-affected herd investigations. The CFT is the primary test for cattle and bison, and the single cervical tuberculin skin test (SCT) and the Dual Path Platform (DPP®) are the primary tests in farmed cervids. Secondary tests are administered to responders. Slaughter surveillance in cattle and bison consists of standardized carcass inspection conducted at federally inspected slaughter establishments (14).

When bTB is suspected, an official investigation is conducted by state and federal animal health regulatory officials (11). This investigation collects information about the premises where the animal resided (city, county, state) and its movements prior to confirming infection. The investigation includes adjacent, contact and possible source herds for the affected herd. Bovine tuberculosis affected herds are classified as epidemiologically linked based on investigative evidence. Investigative evidence includes but is not limited to slaughter establishment records, records of animal movement, such as a bill of sale or certificate of veterinary inspection or other official documents. These records are used to determine where an infected animal resided over time, identify potentially exposed animals and herds and look for the source of infection. Herds exposed through animal movements from a bTB-affected herd are tested, and exposed animals are removed, necropsied and sampled.

### Case definitions

Adult cattle were defined as sexually intact animals >2 years of age, whereas fed cattle are defined as castrated or spayed animals without regard to age that are raised for the purpose of beef production. Another type of cattle are those animals used for roping and other performance events. Castration status has precedence over age, for example, a 4-year-old castrated steer is classified as a fed animal. Slaughter surveillance targets culled adult cattle because these are more likely to exhibit lesions suspicious of bTB (15). bTB-affected herds were classified by production type, including beef, dairy, mixed (beef and dairy), event cattle (roping, rodeo animals), farmed cervids, and unknown.

The case definition used for classifying an animal as confirmed infected with *M. bovis* was either a histologic diagnosis of compatible for mycobacteriosis with a positive polymerase chain reaction (PCR) test performed on formalin fixed tissue using primers for IS*6110* to identify *M. tuberculosis* complex, or bacteriological isolation of *M. bovis*. Affected herds are confirmed when an animal from within the herd is confirmed with bTB. When this criterion cannot be met, the singleton animal (generally found as a result of slaughter surveillance) was defined as a case. The source of infection for affected herds was based on the results of epidemiological investigations as being either unknown, or another USA herd. The latter classification was applied when there was documented animal movement or the potential for fence line or other direct contact. Outbreaks were defined as two or more bTB-affected herds or cases with a documented exposure, such as animal movement between premises.

To identify the likely source, (likely external to the USA or internal transmission within USA) we conservatively estimated that a USA origin strain would not be tlikely to be exported and established in another country after the USA's national bTB prevalence was below 0.5%, which occurred around 1960. Consequently herds that could have shared a common ancestor within the last 60 years would more likely be internal transmission rather than importation. Using the average reported SNP mutation rate of 0.3 SNP/year (16), suggests a reasonable cutoff point of 20 SNPs. Consequently, we considered isolates that were within 20 SNPs of sharing a common ancestor with USA origin cattle to originate from USA and isolates that were within 20 SNPs of sharing a common ancestor with Mexican origin cattle isolates to have originated from Mexico. If more than 20 SNPs had accumulated since sharing a common ancestor with an isolate in the database, we considered the source unknown. Isolates from Michigan cattle within the known endemic area and all Michigan wildlife were excluded.

### Laboratory methods

During slaughter inspection or when bTB test positive animals are euthanized and necropsied, granulomatous-appearing lesions are collected and submitted to the laboratory for histologic examination, PCR testing and mycobacterial culture (17). For some bTB test positive animals, if no visible lesions are observed during necropsy, representative head, abdominal, and thoracic lymph nodes are collected and tested (9, 11, 13). For herds with many bTB infected animals, generally at least 10 isolates were collected and sequenced and in some herds many more were sequenced when sufficient resources existed.

To obtain the WGS, isolates were sequenced on a MiSeq instrument (Illumina, San Diego, CA, United States) using 2 × 250 paired-end chemistry and the Nextera XT library preparation kit (Illumina, San Diego, CA, United States). FASTQ files were put through the NVSL in-house pipeline (https://github.com/USDA-VS). Reads were aligned to the reference genome AF2122/97, NCBI accession number NC_002945 (18), using BWA (19) and post-processing of the alignment was done using Samtools (20). BAM files were processed based on Genome Analysis Toolkit (GATK)'s “best practice” workflow (21, 22). SNPs were called using GATK's HaplotypeCaller with ploidy set to 2, outputting SNPs to variant call format (VCF) files. PPE-PGRS and repeat regions were filtered as well as SNPs that uniformly had QUAL scores < 150 across isolates. To identify SNP calls that were heterozygous, a SNP with an allele call of AC = 1 was relabeled using the International Union of Pure and Applied Chemistry guidelines for ambiguous calls. In order to manage and more accurately analyze this large and diverse dataset, a small number of isolates representing the diversity of the entire dataset were ran through the pipeline. High quality SNPs were identified that clustered the isolates into smaller more manageable groups. Groups were created based on the number of isolates as well as the evolutionary distance. Because these groupings were based on convenience for analysis purposes, they were not necessarily similar in evolutionary distance. For example, group 23 and 24 are very closely related, but because there were so many isolates in that clustered closely together, 2 groups were made. Individual SNPs tables and phylogenetic trees were then created for each group after removing all uninformative SNPs that were homogeneous between the grouped isolates. Because of this process, the reference AF2122, worked as the outgroup isolate for all individual groups. SNPs were then further verified manually using Integrative Genomics Viewer (23) and additional filtering of problematic SNPs was performed on a group by group basis using the SNP tables. Phylogenetic trees were created using RAxML (24) and the GTRCATI model with default settings and accuracy of the phylogenetic tree was confirmed using the manually validated SNP table.

### Data analysis

We examined WGS results for *M. bovis* isolates from cattle and farmed cervid breeding herds and individual animal cases detected through surveillance in the USA, and described the diversity of the isolates within herds and between herds. We identified the most recent common ancestor (MRCA) in the herd. Then, the most closely related isolate was selected from the NVSL WGS database, based on having the fewest number of single nucleotide polymorphisms (SNP) differences, when compared to the herd or case isolate. A pairwise comparison of SNPs to the MRCA was recorded. For herds with *M. bovis* isolates from multiple bTB confirmed infected animals, the tip with the shortest branch length to the tree root was used for the comparison. The most closely related isolates were grouped based on information about the animal or human from which the isolate was obtained. For example, whether the isolate was from imported cattle or a confirmed bTB-affected herd in the USA.

## Results

**Figure 1:**
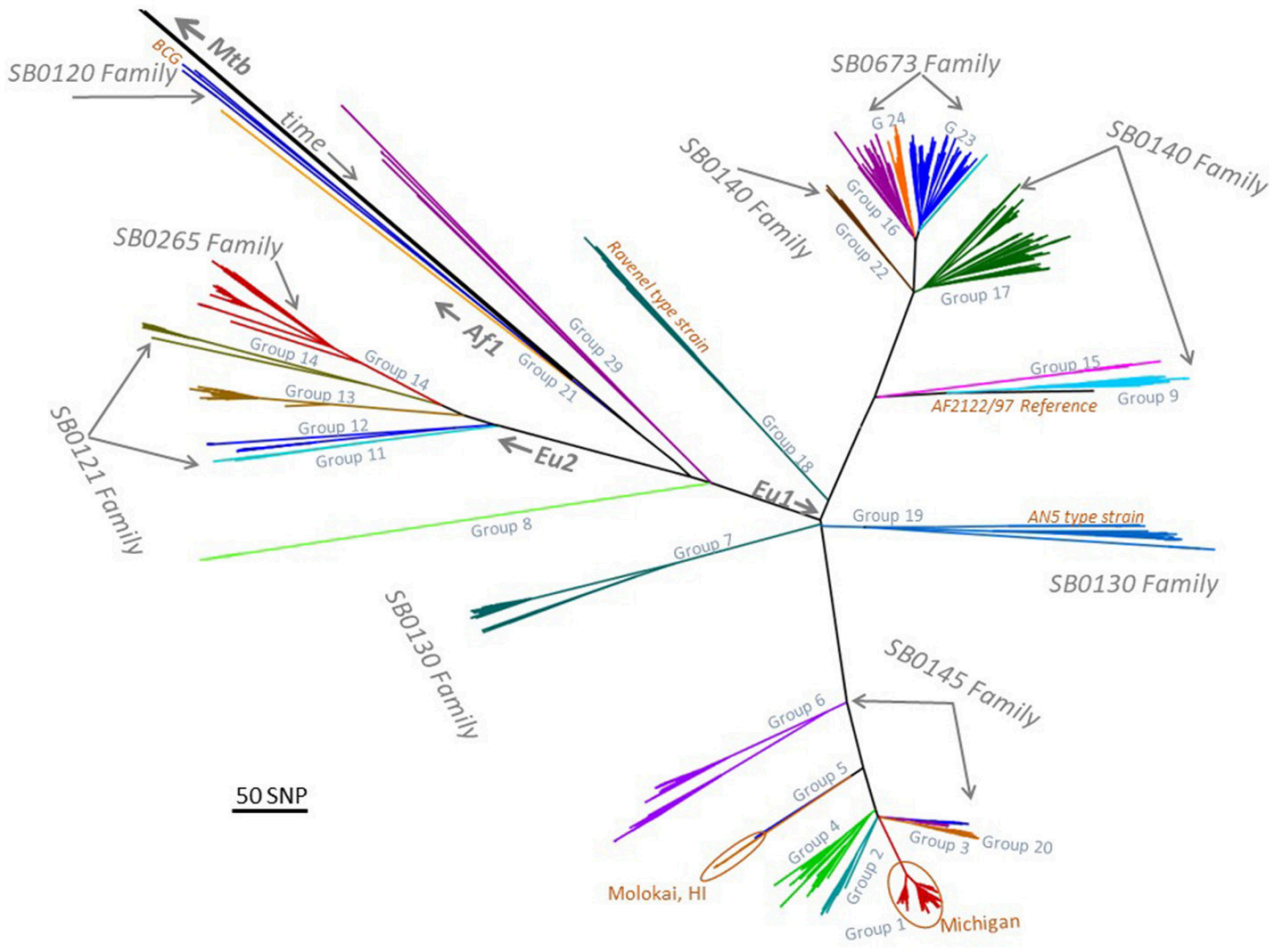
Low resolution phylogenetic tree representing 1,248 *Mycobacterium bovis* isolates and major spoligotyping families. MTBC, M. tuberculosis complex; EU1, European clonal complex 1; EU2, European clonal complex 2; Af1, African complex 1; SNP, single nucleotide polymorphism; BCG, bacille Calmette-Guerin; HI, Hawaii; AN5, *M. bovis* AN5 strain.

Sequencing was performed on 1,248 isolates, this included 154 that were published previously (8, 9). Of these, 185 isolates were collected during 1989–1998, and 1,063 were collected during 1999–2018. The *M. bovis* isolates separated into 24 major phylogenetic groups (Figure 1). Twelve *M. bovis* isolates were obtained from clinical specimens submitted to NVSL (four non-human primates, one jaguar, one elephant, one domestic cat, one brocket deer, 4 unknown species from zoological collections). There were four *M. caprae* isolates from three non-human primates and one rhinoceros residing in zoological collections.

### bTB-affected herds and slaughter cases in culled adult cattle

There were 83 bTB-affected herds, and 26 cases in infected adult domestic cattle during 1999–2018 (Supplemental Table 1). An isolate was not available for two additional cattle herds that are not included in this analysis. The production types included 54 beef herds/cases (49.5%), 37 dairies (33.9%), 3 event/rodeo (2.8%), 3 mixed purpose (2.8%), 11 farmed cervids (10.1%), and one herd of an unknown production type. The herds/cases were located in 21 States (Arizona, California, Colorado, Indiana, Iowa, Kansas, Kentucky, Michigan, Minnesota, Mississippi, North Dakota, Nebraska, New Mexico, New York, Ohio, Oklahoma, Oregon, South Dakota, Texas, Washington, Wisconsin).

There were 563 bTB isolates from these 109 herds/cases (Supplemental Table 1). No herds were identified with more than one strain. Seventy-six (69.7%) of the herds/cases were European clonal complex 1 (EU1) strains, 32 (29.4%) were European clonal complex 2 (EU2) strains, and one herd was infected with a strain not identified within a clonal complex (group 8) (Supplemental Table 1, Table 1). Groups 8 and 14 contain isolates associated with outbreaks in USA and Canada farmed cervid herds (4, 5, 25, 26). The number of isolates per herd ranged from one to 48, (median 2, mean 5), and 52/83 herds (62.7%) had more than one isolate (Supplemental Table 1).

**Table 1:** Whole genome sequencing group for 83 bTB-affected herds and 26 cases during 1999-2018.

**Whole genome sequencing group**	**Number of herds and cases**	**Percent of total**
**EUROPEAN CLONAL COMPLEX 1**		
2	2	1.8
3	1	0.9
4	1	0.9
6	12	11.0
7	11	10.1
16	8	7.3
17	21	19.3
19	1	0.9
20	3	2.8
22	2	1.8
23	13	11.9
**No associated clonal complex**		
8	1	0.9
**EUROPEAN CLONAL COMPLEX 2**		
11	1	0.9
12	2	1.8
13	6	5.5
14	24	22.0
Total	109	100.0

Within herd SNP diversity was evaluated by examining the 18 herds with 10 or more isolates sequenced, identifying the common ancestor, or index sequence, and then counting the number of SNPs accumulated from the index sequence (Table 2). Forty percent of isolates had not diverged or accumulated any SNPs, 86% of the isolates had accumulated 3 or fewer SNPs and no cattle herds contained isolates that had accumulated more than 6 SNPs (Table 2). These accumulated SNPs may be unique to a single isolate or found in a cluster of isolates within a herd. The highest diversity occurred in two herds, a 2009 farmed cervid herd with 28 isolates and 35 SNPs, and a 2017 cattle herd with 25 SNPs among 13 isolates. Based on the epidemiologic investigation, both herds were likely infected for several years.

**Table 2:** The number of single nucleotide polymorphisms (SNPs) from the common ancestor genotypes among bTB-affected herds with >10 isolates, United States, 1999–2018.

	**Number of SNPs**								
Herd name	0	1	2	3	4	5	6	7	8
2001 TX beef (%)	100	0	0	0	0	0	0	0	0
2002 TX dairy (%)	45	27	0	27	0	0	0	0	0
2003 CA dairy (%)	54	23	8	15	0	0	0	0	0
2007 NM dairy (%)	64	29	7	0	0	0	0	0	0
2009 IN cervid A (%)	0	17	0	0	33	33	17	0	0
2009 NE cervid (%)	0	37	11	4	11	15	19	0	4
2010 CO dairy A (%)	83	17	0	0	0	0	0	0	0
2012 CA dairy B (%)	75	25	0	0	0	0	0	0	0
2013 CA dairy (%)	47	27	20	7	0	0	0	0	0
2013 MI Dairy (%)	23	6	64	6	0	0	0	0	0
2014 TX dairy (%)	4	78	6	10	2	0	0	0	0
2015 TX organic dairy (%)	36	28	23	4	6	2	0	0	0
2016 IN beef (%)	13	0	4	29	38	13	4	0	0
2016 IN Longhorn (%)	23	8	31	8	23	0	8	0	0
2016 NM dairy A (%)	19	29	38	10	5	0	0	0	0
2017 NM Dairy A (%)	82	18	0	0	0	0	0	0	0
2017 SD beef A (%)	39	21	6	21	9	3	0	0	0
2017 SD beef D (%)	14	52	29	5	0	0	0	0	0
(%)	**40**	**24**	**14**	**8**	**7**	**4**	**3**	**0**	**0**

*Green, the number of unique SNPs did not occur; Light to dark red, the proportion of isolates that had 0–8 unique SNPs (lower to higher proportion)*.

Despite all of the retrospective data and epidemiological investigations that have been conducted in the USA, we were able to attribute multiple transmission events to a single cow only one time. In this instance, a cow with disseminated TB lesions was sold into a feedlot and exposed a group of cows for <30 days. Within 90 days of exposure, all exposed cows were slaughtered and 6 were identified with lesions. The isolate sequence recovered from a pooled sample of the lesions from the index cow along with the 6 isolate sequences recovered from exposed cows are shown in Table 3. In this event, four different SNP profiles were transmitted to these 6 cows.

**Table 3:** Example of accumulated single nucleotide polymorphisms (SNPs) resulting from multiple transmission events from one animal, 2018.

**Genome position based on the reference NC_002945.4**	**1999500**	**1021045**	**241655**	**1147922**
Reference call	**C**	**C**	**C**	**C**
18-0522_SD_IA_Fed-Cow-Index	S^*^	C	C	C
18-1919_SD_IA_Fed-Cow	**G**	C	C	C
18-1930_SD_IA_Fed-Cow	**G**	C	C	C
18-1932_SD_IA_Fed-Cow	**G**	**T**	C	C
18-1927_SD_IA_Fed-Cow	**G**	**T**	C	C
18-1922_SD_IA_Fed-Cow	C	C	**T**	C
18-1904_SD_IA_Fed-Cow	C	C	C	**T**

* *S Designates a heterogenous SNP call containing both cytosine and guanine. The colors indicate a unique SNP from the reference call*.

The sources of infection based on the investigation results were known for 45/109 (41.3%) of herds/cases (epidemiologically linked via animal movement or adjacent premises contact) and 64 were not epidemiologically linked to another USA origin herd. There was only one documented case of transmission from Mexico, in a roping steer newly imported from Mexico that was found to be infected during a herd test for interstate movement. These 45 herds with a known infection source were associated with 14 outbreaks. Eight outbreaks involved two herds each, two outbreaks involved three herds, one outbreak involved five herds (13), one involved six herds (five herds occurred before 1999) and one outbreak in Minnesota involved 12 herds (8).

The source of infection could not be determined epidemiologically for 64 herds/cases.The most recent common ancestor was within 20 SNPs of a Mexican origin animal for 22 (34.9%) of USA herds/cases suggesting Mexico may be the source (Supplemental Table 1). Twelve cases/herds were within 20 SNPs of unknown origin fed cattle and 20 were within 20 SNPs other USA herds/cases. Isolates from the remaining nine herds/cases, were >20 SNPs from the most recent common ancestor, therefore, no conclusive linkage was found by genetic sequencing or epidemiology. Interestingly, only one of these 64 herds/cases were within 5 SNPs of Mexican origin fed cattle slaughtered in the USA. One additional isolate from a dairy cow was indistinguishable from a worker in the same dairy that was initially diagnosed with bTB, and the dairy was subsequently tested (27).

There was one extensive outbreak involving two closely related clusters within phylogenetic group 14. Group 14 consists of USA farmed cervids and cattle and Canada farmed cervids (4, 5, 25, 26). The first cluster involved 1 farmed cervid herd (Nebraska), three cattle herds [Nebraska, South Dakota (2)], and 1 cattle case in Nebraska during 2009–2013. The cattle herds and case were epidemiologically linked by animal movement or fence line contact and the source herd was a bTB-affected cervid herd [(26), USDA Veterinary Services, unpublished information]. The second cluster, when limited to known animal movement or fence line contact consisted of three cervid (Indiana) and two cattle herds during 2009-2017 (Indiana, Michigan). One of the three Indiana cervid herds (2009) was the source herd. However, three additional cattle herds (Indiana (2), Kentucky) and 3 cases (Indiana (2), Arizona) occurred during 2009-2017, but did not have documented links to the second cluster. Isolates from these six herds/cases are either indistinguishable or have 1-2 SNPs from the 2009 index farmed cervid herd and other group 14 isolates, indicating undocumented animal movements or contacts occurred.

During 1989–1998, there were 50 bTB isolates obtained from 24 affected herds, representing 12 cattle and 12 farmed cervid herds (Supplemental Table 1). These isolates were from 11 States, including Hawaii, Missouri, Montana, New Mexico, New York, Oklahoma, Puerto Rico, Texas, Virginia, Vermont, and Wisconsin. One hundred bTB-affected herds occurred during this time; therefore, isolates were not available for 76 herds. Existing isolates from 1989–1998 separated into 10 major phylogenetic groups (groups 5, 6, 8, 9, 13, 14, 16, 19, 21, 24). Seven of these groups contained isolates recovered from Mexico origin cattle (groups 6, 9, 13, 14, 16, 19, 24), while three did not (groups 5, 8, 21). Four groups were not represented after 1998 (groups 5, 9, 21, 24). The Hawaii isolate was obtained from a beef herd in 1997. The most closely related isolate to the beef herd were from feral swine, obtained during wildlife surveillance efforts there during 2007–2009.

Group 7, subgroup B, provides examples of WGS results (Supplemental File 2). Substantial diversity exists among the 108 isolates, which is typical of the phylogenetic groups. There are two outbreaks, one involving three South Dakota beef herds, and the second involving five Colorado beef and dairy herds [an isolate was not available for one herd, (13)]. For the Colorado outbreak, 12 isolates were sequenced with two unique SNPs; however, the majority of nearly 90 isolates from the index dairy herd were not sequenced due to resource limitations. For the South Dakota outbreak, 30 isolates from the index herd (designated 17-A in the isolate name) were sequenced, with 20 unique SNPs. Isolates from three additional animals sold to other herds (herds 17-B and 17-C) from the index herd had the same SNP pattern as isolates from animals in the index herd. In addition to the two outbreaks, Group 7 includes a single beef herd infection and several single animal cases. The single beef herd occurred in Oklahoma in 2007, for which one fed steer from this herd was found in a Kansas feedlot. There is one case in an adult beef cow found through slaughter surveillance that traced to New Mexico in 2004. This case could not be linked to a bTB-affected herd. The isolate from this animal is only one SNP different from the 2007 Oklahoma herd. It is hypothesized the 2004 case originated from the Oklahoma herd; however, investigative information is no longer available. A second, unrelated case found through slaughter surveillance occurred during 2005 in a Nebraska beef cow, in which bTB could not be confirmed in the herd of origin. The most closely related isolates to this beef cow are from unknown (1992) and Mexican (2012) origin fed cattle slaughtered in the US (10 and 12 SNPs, respectively). Other group 7 isolates include 36 cases in fed cattle from 1991–2012; many of these were imported from Mexico and slaughtered in Texas. Finally, there are 18 results for isolates from cattle in Mexico.

### Slaughter cases in fed cattle

**Figure 2:**
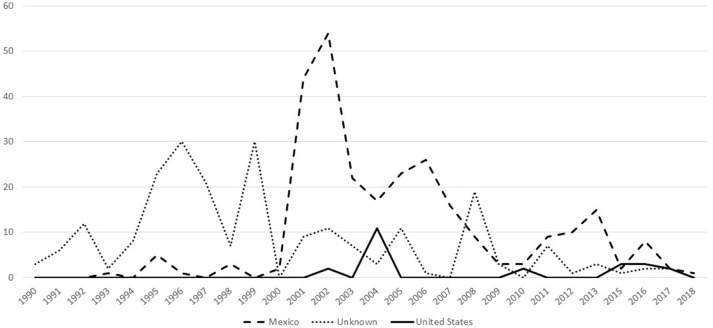
Number of bTB cases that have whole genome sequencing results, by year and country of animal's origin for fed cattle slaughtered in the U.S, federal fiscal years 1990-2018 (*n* = 521).

There were a total of 521 confirmed bTB cases in fed cattle found through slaughter inspection during 1990–2018 (Figure 2). These isolates separated into 19 phylogenetic groups (Table 4). One isolate had a mixed infection. When considered by country of origin, 276 (53.0%) were from Mexico, the country of origin could not be determined for 223 (42.8%) and 22 (4.2%) occurred in USA origin cattle. An additional two cases in fed cattle from Canada slaughtered in Washington State were classified in group 23 (data not shown). Twenty of the USA origin cases separated into six phylogenetic groups and originated from six known bTB-affected herds. One case was untraceable and one case was under investigation at the time of this report. The country of origin could not be determined for 223 isolates from fed cattle. The most common reason that country of origin cannot be determined is because official animal identification was not available and the infected animal had been comingled with both USA and Mexican origin cattle in pastures or feedlots prior to slaughter (VS unpublished). Twenty-four isolates (4.6%) were from cases that occurred during 1989–1993, representing a small fraction of 1,504 bTB feedlot investigations that were reported during 1989–1993 (28).

**Table 4:** Number of isolates by whole genome sequencing group and country of animal's origin for fed cattle slaughtered in the U.S, 1990–2018.

**Group**	**Mexico**	**Unknown**	**USA**	**Total**
Mixed	0	1	0	1
2	6	3	0	9
3	1	0	0	1
4	7	7	0	14
6	15	10	1	26
7	24	21	0	45
9	7	1	0	8
11	1	0	0	1
12	3	2	0	5
13	17	23	1	41
14	8	3	6	17
15	5	9	0	14
16	17	12	4	33
17	49	69	0	118
18	2	0	0	2
19	4	1	0	5
20	1	2	0	3
22	9	7	0	16
23	83	46	10	139
24	17	6	0	23
Total	276	223	22	521

Four groups (9, 15, 18, 24) were represented in fed cattle cases but not bTB-affected herds and cases, and one group was found in a farmed cervid herd but not in fed cattle (group 8). The five largest groups are 7, 13, 16, 17, and 23, and contain 72.3% of isolates from fed cattle. In comparison, the five largest groups are 6, 7, 14, 17, and 23, containing 65.4% of affected herds and cases during 1989–2018.

### Spoligotyping

A comparison of spoligotyping and whole genome sequencing results are shown in Figure 1. There are six spoligotyping families that occur in the USA and each WGS group falls within one spoligotype family. Spoligotyping family SB0673 contains WGS Groups 16, 23, and 24; SB0120 contains Group 21; SB0121 contains Groups 11-14; SB0140 contains Groups 9, 17, 17 and 22; SB0130 contains Groups 7 and 19; and, SB0145 contains Groups 1-6 (Figure 1).

## Discussion

Consistent with previous reports, broad diversity exists among USA bTB isolates detected in cattle and farmed cervids (7). Not unexpectedly, many of the fed cattle isolates were from imported cattle, and contain even more diversity. WGS results do not support a bTB reservoir in USA cattle. The bTB eradication program appears to be highly effective as the majority of herds/cases in the USA are unique strains with limited herd to herd transmission. Two major exceptions occurred outside the endemic area of Michigan: the first in farmed cervid herds that subsequently spilled into cattle herds (15 herds/cases from 2009 to 2018) and the second in Minnesota where bTB spilled over into the local white-tailed deer and 12 cattle herds were affected (8). Farmed cervids are subject to official bTB program requirements including surveillance, and no bTB-affected cervid herds have occurred since 2009.

In all cases, WGS results corroborated investigative evidence of herd-to-herd transmission events. Not unexpectedly, there was less SNP diversity between epidemiologically linked herds, almost half these herds had no unique SNPs and the maximum number of unique SNPs was four (Supplemental Table 1). Nearly half of the herd to herd transmission events had a SNP genotype that had been found in the source herd.

WGS results also discovered previously unrecognized links between herds and cases. Isolates from 12 herds/cases with an unknown source of infection were within three SNPs of other USA herds/cases during 2009–2018, including six herds/cases clustering within the group 14 outbreak. In another example, the isolate from a 2010 bTB-infected Holstein cow from an Ohio dairy had one additional SNP from a single isolate obtained from a 2008 New Mexico dairy cow (Supplemental Table 1). Investigative evidence indicated the 2010 Ohio dairy cow originated from New Mexico though no direct links could be found to the 2008 herd. These findings raise the possibility of undocumented animal movements or exposure. A pathways analysis of 12 bTB-affected California dairy herds based predominantly on herd investigations, concluded that with one exception, *M. bovis* occurred because of independent introductions from sources outside the system (12).

Most of the isolates recovered from imported fed cattle are not closely related to isolates from USA herds, with only one of the 109 herds/cases within 5 SNPs of an imported fed steer, despite having nearly complete representation of fed cattle and affected herds during 1999–2018. This suggests there may be other vectors transmitting bTB to the USA national herd such as humans or even imported dairy products (29, 30). It may also be possible to have undetected residual strains from historical cases. In one example, 11 years elapsed between bTB detection in epidemiologically linked California dairy herds (12). Alternatively, a limitation of this analysis are missing isolates from bTB-infected animals not detected through surveillance activities. Slaughter surveillance in the USA has an estimated sensitivity of detecting a bTB affected herd in 1 year of 3.2% in small beef herds (1-49 head), to 50.6% in large dairies (>500 head) (31). A Bayesian molecular clock phylogenetic analysis of the Minnesota outbreak reported the median time to the most recent common ancestor was 1999 (range 1991, 2005) for isolates from the Minnesota outbreak (3 SNPs) and its mostly closely related isolate, a 2012 Texas beef herd (8 SNPs) (8). We were unable to determine if there were two introductions into the USA of a closely related genotype from Mexico, or if common ancestors were present in the USA, possibly as early as 1991, but were not present in the NVSL database.

The most closely related isolates to some affected herds are from fed cattle that occurred earlier in time than the affected herds, many prior to 1999. We hypothesized that if imported cattle were the source of infection for affected herds, we would find closely related isolates among imported fed cattle and affected herds/cases during 1999–2018. That pre-1999 fed cattle cases are the closest match to some post-1999 affected herds/cases is noteworthy, because the pre-1999 isolates from fed cattle represent only a small fraction of the cases that occurred (28). There was a substantially higher risk of bTB introduction from Mexican origin cattle during 1983–1993, compared to today (28).

Limited diversity occurred within most herds, with the majority of isolates having three or fewer SNPs. This may be useful in guiding epidemiologic investigations. For example, additional unique SNPs may indicate that an intermediary herd exists. The estimated SNP occurrence per genome per year ranges from 0.147 (32) to 0.53 (2.5% lower 0.22, 97.5% upper 0.94, (16). Applying these to the time frames and SNPs reported here may indicate a common exposure for the isolates with five or fewer SNPs, while separate introduction events may be more likely for isolates with >5 SNPs. The unique SNPs observed among animals within a herd may represent strain variation caused by animal to animal transmission, or an actual mutation. In the one example we observed, four different SNP profiles were transmitted to six cows exposed for 30 days to a single bTB-infected beef cow. Thacker et al. (33) reported that a single SNP genotype was recovered in 80% of affected tissues from experimentally infected white tailed deer. The inoculum and isolates from the remaining animals contained different WGS genotypes, some with the same SNP, and it was hypothesized that the SNP was not a mutation but was present though undetected in the original inoculation.

The use of WGS in new cases has focused epidemiological investigations and significantly reduced time and costs associated with these investigations and reduced the burden on livestock producers. Whole genome sequencing results for a 2013 case in a California Holstein heifer without animal identification enabled resources to focus on testing an epidemiologically linked herd (Supplemental Table 1). If these results had not been available, as many as 60 herds that had contributed cattle to the slaughter lot of the bTB infected case would have been administered a whole herd test. However, care must also be taken that the scope of the investigation not be prematurely limited by WGS results, at the risk of missing cases. WGS results provided critical information that enabled the successful detection of bTB spread to cattle from the 2009 NE infected farmed cervid herd (26). In another case, whole genome sequencing evidence linked three bTB-affected cattle operations to the index herd in the absence of cattle movements between the premises (9).

bTB prevalence in dairies is approximately twice that of beef herds (3). The risk factors for disease transmission among beef and dairy herds in the USA are unknown. The WGS results reported here may be useful in guiding future work to identify risk factors for bTB transmission. General trends in the beef and dairy industries during the study period include a decrease in the number of beef and dairy cattle and cattle operations during 1993–2008, while the average herd size has increased (34–36). The magnitude of this change was greater in dairy operations, where the number of operations declined 58.4% during 1991–2006, while the average dairy herd size doubled from 54 to 122 cows (36). Larger dairies continue to increase in herd size, and almost 30% of dairy operations introduced new cattle. Similarly, almost 35% of cow-calf beef operations introduced new cattle, most commonly weaned beef bulls and weaned steers (34).

Transmission from human workers to cattle has been hypothesized, especially for dairy cattle operations because of intensive management practices (12). In 2013, a USA dairy worker was diagnosed with bTB. The dairy herd was tested and bTB was confirmed in three animals (27). One animal was infected with the identical strain as the worker. While the direction of transmission could not be determined, this case suggests the possibility of human to animal transmission, as no other sources of exposure for the cattle could be identified. An evaluation of human and cattle *M. bovis* isolates from Baja California, Mexico found that 155 isolates from cattle and 17 from humans clustered into seven major groups (37). The human isolates were interspersed among the cattle isolates, and cheese is the suspected source of exposure of *M. bovis* for humans. In these examples from the USA and Mexico, direct or indirect contact between the human and animal subjects occurred; however, as noted previously, closely related isolates should not be used to imply transmission events, in the absence of epidemiologic linkages.

All isolates were sequenced for some herds, particularly those with a small number of bTB infected animals, but because of resource limitations, a smaller proportion of available isolates were sequenced for larger herds with dozens or hundreds of bTB infected animals. This limitation may bias these results by potentially under reporting the number of SNPs in herds with large numbers of infected animals, or conversely, fail to document if limited SNPs are present despite extensive within herd transmission.

## Conclusion

Whole genome sequencing has become a cost effective, essential tool for the USA official bTB eradication program, providing information that increases the success and efficiency of the extensive investigations that occur when bTB is confirmed in cattle and farmed cervids. The isolates that occur among USA livestock are diverse, and the lack of diversity within herds support that a reservoir does not exist in the USA cattle population, although transmission of one strain has continued to occur from farmed cervids to cattle herds. The source of infection is unknown for approximately half of bTB-affected herds, and WGS results suggest several possible sources, including undocumented cattle movement, imported cattle, as well as humans or unpasteurized dairy products.

## Author contributions

SR-A and TS performed WGS and provided summarized results. KO and SR-A compiled and analyzed the summarized WGS results. KO and SR-A wrote the paper. BH and MS provided epidemiological information. BH and MS reviewed/edited the paper.

### Conflict of interest statement

The authors declare that the research was conducted in the absence of any commercial or financial relationships that could be construed as a potential conflict of interest. The reviewer AD and the handling Editor declared their shared affiliation.
